# Supplementary material for: Knowledge, attitude, and practice on antibiotic use and antibiotic resistance among the veterinarians and para-veterinarians in Bhutan
Source: PLoS One. 2021 May 6;16(5):e0251327. doi: 10.1371/journal.pone.0251327 (PMC8101766; doi:10.1371/journal.pone.0251327)
Supplement: S1 Table — (DOCX) [file pone.0251327.s001.docx]

**S1 Table.** **Bivariate analysis of knowledge on antibiotics and AMR among the veterinarian and para-veterinarians categorized by the level of qualification**

| **Characteristic** | **Variables/Categories** | **Frequency (%)** | **Certificate/Diploma (%)** | **Bachelor/Masters (%)** | **p-value** |
| --- | --- | --- | --- | --- | --- |
| Antibiotic are prescribed for | |  |  |  |  |
|  | Bacterial infection | 162 (75) | 118 (75.2) | 44 (74.6) | 0.93 |
|  | Incorrect answer/others | 54 (25) | 39 (24.8) | 15 (25.4) |  |
| Antibiotics should be administered with correct dose and dosage for an animal species | |  |  |  |  |
|  | Yes | 219 (100) | 160 (100) | 59 (100) |  |
|  | No | 0 | 0 | 0 |  |
| The antibiotic treatment should be stopped once the animal stops showing signs of disease even if the course is not completed | |  |  |  |  |
|  | No | 208 (95.4) | 151 (95) | 57 (96.6) | 1.00** |
|  | Incorrect answer | 10 (4.6) | 8 (5) | 2 (3.4) |  |
| Giving antibiotics to animals that are not sick will prevent it from becoming sick in the future. | |  |  |  |  |
|  | No | 200 (91.3) | 146 (91.3) | 54 (91.5) | 0.949 |
|  | Incorrect answer | 19 (8.7) | 14 (8.8) | 5 (8.5) |  |
| If one animal in a herd is sick, all other animals in the same herd should be given antibiotics to prevent infection | |  |  |  |  |
|  | No | 197 (90) | 145 (90.6) | 52 (88.1) | 0.587 |
|  | Incorrect answer | 22 (10) | 15 (9.4) | 7 (11.9) |  |
| Antibiotics should be given to promote growth in animals | |  |  |  |  |
|  | No | 208 (95) | 152 (95) | 56 (94.9) | 1.00** |
|  | Incorrect answer | 11 (5) | 8 (5) | 3 (5.1) |  |
| Broilers treated with antibiotics should NOT be slaughtered for meat purpose until the completion of withdrawal period of that antibiotic. | |  |  |  |  |
|  | Yes | 212 (96.8) | 154 (96.3) | 58 (98.3) | 0.677** |
|  | Incorrect answer | 7 (3.2) | 6 (3.8) | 1 (1.7) |  |
| Milk and milk products from a cow treated with antibiotics can be consumed during the course of treatment | |  |  |  |  |
|  | No | 206 (94.1) | 149 (93.1) | 57 (96.6) | 0.521** |
|  | Incorrect answer | 13 (5.9) | 11 (6.9) | 2 (3.4) |  |
| What is the average duration of antibiotic course in large animal? | |  |  |  |  |
|  | 5-7 days | 169 (78.2) | 122 (77.2) | 47 (81) | 0.547 |
|  | Incorrect answer | 47 (21.8) | 36 (22.8) | 11 (19) |  |
| What is the average duration of antibiotic course in poultry birds | |  |  |  |  |
|  | 5-7 days | 107 (50.7) | 73 (46.2) | 34 (57.6) | 0.134 |
|  | Incorrect answer | 110 (49.3) | 85 (53.8) | 25 (42.4) |  |
| Any bacteria will become resistant once it is exposed to an antibiotic | |  |  |  |  |
|  | Yes | 122 (66) | 93 (58.5) | 29 (49.2) | 0.217 |
|  | Incorrect answer | 96 (44) | 66 (41.5) | 30 (50.8) |  |
| An animal infected with resistant bacteria will be difficult to treat. | |  |  |  |  |
|  | Yes | 209 (95.4) | 151 (94.4) | 58 (98.3) | 0.294 |
|  | Incorrect answer | 10 (4.6) | 9 (5.6) | 1 (1.7) |  |
| A resistant bacterium can be spread between animals and also to humans | |  |  |  |  |
|  | Yes | 127 (58) | 80 (50) | 47 (79.7) | <0.001 |
|  | Incorrect answer | 92 (42) | 80 (50) | 12 (20.3) |  |
| Practicing good animal hygiene will prevent development of AMR | |  |  |  |  |
|  | Yes | 119 (54.3) | 88 (55) | 31 (52.5) | 0.746 |
|  | Incorrect answer | 100 (45.7) | 72 (45) | 28 (47.5) |  |
| A cow with a recurrent mastitis is brought to your clinics. The cow was previously treated with penicillin intramammary infusion. What antibiotics will you prescribe now? | |  |  |  |  |
|  | Conduct AST on milk samples | 77 (35.5) | 52 (32.7) | 25 (43.1) | 0.157 |
|  | Incorrect answer | 140 (64.5) | 107 (67.3) | 33 (56.9) |  |
| Three birds in a flock of 500 showed signs of greenish diarrhoea. The birds are eating normal and active. What antibiotics will you prescribe? | |  |  |  |  |
|  | No need for any antibiotics | 25 (11.5) | 13 (8.2) | 12 (20.7) | 0.011 |
|  | Incorrect answer | 192 (88.5) | 146 (91.8) | 46 (79.3) |  |
| An owner complaint of his bull having fever and inappetence for three days. There is salivation but mucus membranes are pale pink. What antibiotic will you prescribe? | |  |  |  |  |
|  | No need for any antibiotics | 72 (35.1) | 49 (32.7) | 23 (41.8) | 0.224 |
|  | Incorrect answer | 133 (64.9) | 101 (67.3) | 32 (58.2) |  |
