# Supplementary material for: Knowledge, attitude, and practice on antibiotic use and antibiotic resistance among the veterinarians and para-veterinarians in Bhutan
Source: PLoS One. 2021 May 6;16(5):e0251327. doi: 10.1371/journal.pone.0251327 (PMC8101766; doi:10.1371/journal.pone.0251327)
Supplement: S2 Table — (DOCX) [file pone.0251327.s002.docx]

**S2 Table. Attitude towards antibiotics and AMR among the veterinarian and para-veterinarians in Bhutan**

| **Sl No** | **Questions** | **Strongly agree (%)** | **Agree n, %)** | **Neutral (n, %)** | **Disagree (n, %)** | **Strongly disagree (n, %)** |
| --- | --- | --- | --- | --- | --- | --- |
| 1 | Thorough examination of animals is required before prescribing or administering an antibiotic | 69.4 | 26.9 | 2.7 | 0.5 | 0.5 |
| 2 | Antibiotic susceptibility test is required before administering antibiotics | 26.9 | 48.4 | 19.6 | 4.6 | 0.5 |
| 3 | Broad spectrum antibiotics are correct choice for any bacterial infections | 18.7 | 53.4 | 12.8 | 11.4 | 3.67 |
| 4 | Antibiotic resistance is a serious problem in the world | 61.6 | 34.7 | 2.7 | 0.9 | 0 |
| 5 | Antibiotic resistance in animal is a problem in Bhutan | 13.2 | 46.6 | 32 | 8.2 | 0 |
| 6 | Antibiotic resistance is a result of inappropriate use of antibiotics | 51.1 | 37 | 6.9 | 4.6 | 0.5 |
| 7 | Treating animals with inappropriate antibiotics (abusing antibiotic use in animals) will develop antibiotic resistance in human | 32.9 | 42 | 14.2 | 9.1 | 1.8 |
| 8 | Treatment failure of bacterial infection is due to development of antibiotic resistance | 30.1 | 49.3 | 16 | 4.1 | 0.5 |
| 9 | Antibiotic resistance is not an issue as there are always new antibiotics being discovered and available in the market | 3.2 | 6.4 | 18.7 | 47 | 24.7 |
| 10 | Administration of correct dose and dosage of antibiotics can prevent development of resistance. | 54.8 | 37 | 3.7 | 4.1 | 0.5 |
| 11 | Antibiotic resistance will affect me, my family’s health or everyone’s health | 48.4 | 40.2 | 6.9 | 3.2 | 1.4 |
