# Supplementary material for: Knowledge, attitude, and practice on antibiotic use and antibiotic resistance among the veterinarians and para-veterinarians in Bhutan
Source: PLoS One. 2021 May 6;16(5):e0251327. doi: 10.1371/journal.pone.0251327 (PMC8101766; doi:10.1371/journal.pone.0251327)
Supplement: S3 Table — (DOCX) [file pone.0251327.s003.docx]

**S3 Table. Prescribing pattern of antibiotics among the veterinarian and para-veterinarians in Bhutan**

| Characteristic | Frequency (%) | Certificate/diploma (%) | Bachelor/Masters (%) | p-value |
| --- | --- | --- | --- | --- |
| **How often do you prescribe (or give/administer) antibiotics?** | | | |  |
| Daily | 41(19) | 25 (15.8) | 16 (27.6) | 0.13** |
| More than once in a week | 106 (49.1) | 77 (48.7) | 29 (50) |  |
| Less than once a month | 37(17.1) | 31 (19.6) | 6 (10.3) |  |
| More than once a month | 32(14.8) | 25 (15.8) | 7 (12.1) |  |
| **How confident are you in prescribing antibiotics for animal treatment?** | | | |  |
| Not confident at all | 2(0.9) | 2 (1.3) | 0 | 0.276 |
| Slightly confident | 22 (10.1) | 13 (8.1) | 9 (15.3) |  |
| Somewhat confident | 63(28.8) | 49 (30.6) | 14 (23.7) |  |
| Fairly confident | 100(45.7) | 75 (46.9) | 25 (42.4) |  |
| Completely confident | 32(14.6) | 21 (13.1) | 11 (18.6) |  |
| **I can calculate antibiotic dose and dosage for a particular species of animal** | | | |  |
| Not confident at all | 4(1.8) | 4 (2.5) | 0 | 0.064 |
| Slightly confident | 20 (9.1) | 16 (10 ) | 4 (6.8) |  |
| Somewhat confident | 46 (21) | 35 (21.9) | 11 (18.6) |  |
| Fairly confident | 88 (40.2) | 69 (43.1) | 19 (32.2) |  |
| Completely confident | 61 (27.9) | 36 (22.5) | 25 (42.4) |  |
| **I prescribe or administer antibiotics based on the availability in my center** | | | |  |
| Yes | 151 (69.3) | 109 (68.6) | 42 (71.2) | 0.708 |
| No | 67 (30.7) | 50 (31.5) | 17 (29.8) |  |
| **Do you use any guidelines/manual /text books for prescribing antibiotics?** | | | |  |
| Yes | 208 (95.4) | 153 (95.6) | 55 (94.8) | 0.728 |
| No | 10 (4.6) | 7 (4.4) | 3 (5.2) |  |
| **Do you seek expert (veterinarian/ specialist) advice before prescribing antibiotics?** | | | |  |
| Yes | 178 (82) | 136 (85.5) | 42 (72.4) | 0.026** |
| No | 39 (18) | 23 (14.5) | 16 (27.6) |  |
| **Do you use expired antibiotics in the absence of viable antibiotics in your center?** | | | |  |
| Yes | 27 (12.3) | 22 (13.8) | 5 (8.5) | 0.292 |
| No | 192 (87.7) | 138 (86.2) | 54 (91.5) |  |
| **Do you face shortage of antibiotics in your center?** | | | |  |
| Yes | 102 (46.6) | 67 (41.9) | 35 (59.3) | 0.022** |
| No | 117 (53.4) | 93 (58.1) | 24 (40.7) |  |
| **Drug expiry is a problem in my center** | | | |  |
| Yes | 71 (32.6) | 53 (33.1) | 18 (31) | 0.771** |
| No | 147 (67.4) | 107 (66.9) | 40 (69) |  |
| **How do you decide upon whether or not to prescribe an antibiotic for a case?** | | | |  |
| Based on the previous experience | 153 (71.2) | 111 (70.3) | 42 (73.7) | 0.804 |
| After consultation with other professionals | 61 (28.4) | 46 (29.1) | 15 (26.3) |  |
| Upon farmers’/owner’s request | 1 (0.5) | 1 (0.6) | 0 |  |
