# Supplementary material for: Knowledge, attitude, and practice on antibiotic use and antibiotic resistance among the veterinarians and para-veterinarians in Bhutan
Source: PLoS One. 2021 May 6;16(5):e0251327. doi: 10.1371/journal.pone.0251327 (PMC8101766; doi:10.1371/journal.pone.0251327)
Supplement: S1 Text — (PDF) [file pone.0251327.s004.pdf]

KAP survey on antibiotics and AMR

Kuzuzangpola!, This is Dr. Karma Wangmo working as senior veterinary officer in RLDC, Khagma. We are conducting a survey to understand the knowledge, perception and practice on antibiotic use and antibiotic resistance (AMR) among the livestock staffs in Bhutan. The study has been approved by the Research and Extension Division, DoL. Your response will immensely help in making a policy decision on the usage of antibiotics in animals and AMR in Bhutan. The survey will take only about 10-12 minutes and your response will be recorded as anonymous. Please, kindly spare a few minutes and give your valuable response by clicking the link below. Thank you!

Respondent's information

Please tick /circle the appropriate answer(s) and specify wherever required

1. 101. What is your gender?

Mark only one oval.

- Male
- Female

2. 102. What is your age?

Mark only one oval.

- 20-30 years
- 31-40 years
- 41-50 years
- more than 50 years

3. 103. What is your level of qualification in relation to veterinary/animal sciences?

Mark only one oval.

- Certificate
- Diploma
- Bachelor
- Masters
- PhD

4. 104. Which offices/organization are you currently working?

Mark only one oval.

- Gewog Extension Center
- Dzongkhag Veterinary Hospital
- Thromde Veterinary Hospital
- National Veterinary Hospital

5. 105. Which Dzongkhag are you currently working?

Mark only one oval.

- Thimphu
- Paro
- Haa
- Chukha
- Samtse
- Gasa
- Punakha
- Wangdue
- Tsirang
- Dagana
- Sarpang
- Zhemgang
- Trongsa
- Bumthang
- Lhuentse
- Mongar
- Trashigang
- Trashiyangtse
- Pemagatshel
- Samdrup Jongkhar

6. 106. What is your current position?

Mark only one oval.

- Veterinarian
- Animal Health supervisor
- Livestock Production Supervisor
- Extension Supervisor
- Livestock Production Officer
- Geog Livestock Extension Officer

7. 107. How many years have you been working in the government /livestock sector?

Mark only one oval.

- 0-5 years
- 6-10 years
- 11-20 years
- more than 20 years

Questions related to knowledge on Antibiotics and Antimicrobial Resistance (AMR)

Please tick /circle the appropriate answer(s) and specify wherever required

8. 201. What is an antibiotic prescribed for?

Mark only one oval.

- Viral infection
- Bacterial infection
- Fungal infection
- Protozoal infection
- All the above

9. 202. Antibiotics should be administered with correct dose and dosage for an animal species

Mark only one oval.

- Yes
- No
- Don’t know

10. 203. The antibiotic treatment should be stopped once the animal stops showing signs of disease even if the treatment course is not completed.

Mark only one oval.

- Yes
- No
- Don’t know

11. 204. Giving antibiotics to animals that are not sick will prevent it from becoming sick in the future

Mark only one oval.

- Yes
- No
- Don’t know

12. 205. If one animal in a herd is sick, all other animals in the same herd should be given antibiotics to prevent infection

Mark only one oval.

- Yes
- No
- Don’t know

13. 206. Antibiotics should be given to promote growth in animals

Mark only one oval.

- Yes
- No
- Don’t know

14. 207. Broilers treated with antibiotics should NOT be slaughtered for meat purpose until the completion of withdrawal period of that antibiotic.

Mark only one oval.

- Yes
- No
- Don’t know

15. 208. Milk and milk products from a cow treated with antibiotics can be consumed during the course of treatment

Mark only one oval.

- Yes
- No
- Don’t know

16. 209. What is the average duration of antibiotic course in large animal?

Mark only one oval.

- 1 day
- 3 days
- 5 days
- 7 days
- 10 days
- Don't know

17. 210. What is the average duration of antibiotic course in poultry birds?

Mark only one oval.

- 1 day
- 3 days
- 5 days
- 7 days
- 10 days
- Don't know

18. 211. Have you heard the term Antimicrobial resistance (AMR)?

Mark only one oval.

- Yes
- No

19. 212. If, yes, from where did you hear about AMR? (you can select more than one)

Check all that apply.

- [ ] Radio
- [ ] TV
- [ ] News Paper
- [ ] Internet/Social Media
- [ ] Training program
- [ ] Awareness program/campaign

20. 213. Any bacteria will become resistant once it is exposed to an antibiotic

Mark only one oval.

- Yes
- No
- Don’t know

21. 214. An animal infected with resistant bacteria will be difficult to treat.

Mark only one oval.

- Yes
- No
- Don't know

22. 215. A resistant bacterium can be spread between animals and also to humans

Mark only one oval.

- Yes
- No
- Don't know

23. 216. Good management practice will prevent development of AMR

Mark only one oval.

- Yes
- No
- Don't know

24. 217. Have you read Bhutan National Plan on AMR?

Mark only one oval.

- Yes
- No

25. 218. Have you heard/participated in Antibiotic Awareness Week?

Mark only one oval.

- Yes
- No

26. 219. Have you attended any refresher course/training on usage of antibiotics

Mark only one oval.

- Yes
- No

27. 220. A cow with a recurrent mastitis is brought to your clinics. The cow was previously treated with penicillin intramammary infusion. What antibiotics will you prescribe now?

Mark only one oval.

- Penicillin intramammary infusion
- Gentamicin injection
- Cefoperazone intramammary
- Conduct antibiotic sensitivity test on milk samples
- No need antibiotics

28. 221. Three birds in a flock of 500 showed signs of greenish diarrhea. The birds are eating normal and active. What antibiotics will you prescribe?

Mark only one oval.

- Tetracycline for all the birds
- Tetracycline for the three sick birds
- Sulphadiazine for all the birds
- Sulphadiazine for the three sick birds
- No need for any antibiotics

29. 222. An owner complaint of his bull having fever and inappetence for three days. There is salivation but mucus membranes are pale pink. What antibiotic will you prescribe?

Mark only one oval.

- Oxytetracycline
- Amoxycillin
- Gentamicin
- Sulphadimidine and trimethoprim combination
- Streptopenicillin.
- No need any antibiotics.

Questions related to Perceptions on use of antibiotics and AMR

Please tick /circle the appropriate answer(s) and specify wherever required

30. 301. Thorough examination of animals is required before prescribing or administering an antibiotic

Mark only one oval.

- Strongly agree
- Agree
- Neutral
- Disagree
- Strongly disagree

31. 302. Antibiotic susceptibility test is required before administering antibiotics

Mark only one oval.

- Strongly agree
- Agree
- Neutral
- Disagree
- Strongly disagree

32. 303. Broad spectrum antibiotics are correct choice for any bacterial infections

Mark only one oval.

- Strongly agree
- Agree
- Neutral
- Disagree
- Strongly disagree

33. 304. Antibiotic resistance is a serious problem in the world

Mark only one oval.

- Strongly agree
- Agree
- Neutral
- Disagree
- Strongly disagree

34. 305. Antibiotic resistance in animal is a problem in Bhutan

Mark only one oval.

- Strongly agree
- Agree
- Neutral
- Disagree
- Strongly disagree

35. 306. Antibiotic resistance is a result of inappropriate use of antibiotics

Mark only one oval.

- Strongly agree
- Agree
- Neutral
- Disagree
- Strongly disagree

36. 307. Treating animals with inappropriate antibiotics (abusing antibiotic use in animals) will develop antibiotic resistance in human

Mark only one oval.

- Strongly agree
- Agree
- Neutral
- Disagree
- Strongly disagree

37. 308. Failure of antibiotic therapy is due to development of antibiotic resistance

Mark only one oval.

- Strongly agree
- Agree
- Neutral
- Disagree
- Strongly disagree

38. 309. Antibiotic resistance is not an issue as there are always new antibiotics being discovered and available in the market

Mark only one oval.

- Strongly agree
- Agree
- Neutral
- Disagree
- Strongly disagree

39. 310. Administration of correct dose and dosage of antibiotics can prevent development of resistance.

Mark only one oval.

- Strongly agree
- Agree
- Neutral
- Disagree
- Strongly disagree

40. 311. Antibiotic resistance will affect me, my family's health or everyone's health

Mark only one oval.

- Strongly agree
- Agree
- Neutral
- Disagree
- Strongly disagree

Questions related to Practices and prescribing pattern on antibiotics and AMR

Please tick /circle the appropriate answer(s) and specify wherever required

41. 401. What type of animal cases do you prescribe (give/administer) antibiotics?

Mark only one oval.

- Any type of cases
- Animal with rhinitis
- Animal with diarrhea
- Animal with inappetence
- Prolong illness with underlying bacterial infection

42. 402. How often do you prescribe (or give/administer) antibiotics?

Mark only one oval.

- Daily
- More than once in a week
- Less than once a month
- More than once a month

43. 403. I make my own judgement to prescribe or administer antibiotics to animals and is not influenced by the animal owner’s decision (not based on what owners want)

Mark only one oval.

- Yes
- No

44. 404. How confident are you in prescribing antibiotics for animal treatment?

Mark only one oval.

- Not confident at all
- Slightly confident
- Somewhat confident
- Fairly confident
- Completely confident

45. 405. I can calculate antibiotic dose and dosage for a particular species of animal

Mark only one oval.

- Not confident at all
- Slightly confident
- Somewhat confident
- Fairly confident
- Completely confident

46. 406. How many days do you usually prescribe (duration of antibiotic treatment) antibiotics for an animal?

Mark only one oval.

- One day
- Three days
- Five days
- Seven days
- Until the animal recovers

47. 407. I think of the type of bacteria involved in infection of an animal before selecting an antibiotic

Mark only one oval.

- Yes
- No

48. 408. I prescribe or administer antibiotics based on the availability in my center

Mark only one oval.

- Yes
- No

49. 409. Which antibiotics is most commonly used/prescribed by you in your center? (You can choose more than one)

Check all that apply.

- [ ] Oxytetracycline
- [ ] Benzathin penicillin
- [ ] Ampicillin cloxacillin
- [ ] Streptopencillin
- [ ] Gentamicin
- [ ] Tetracycline
- [ ] Sulphonamides
- [ ] Cefotaxime

50. 410. Do you use any guidelines/manual /text books for prescribing antibiotics?

Mark only one oval.

- Yes
- No

51. 411. Do you seek expert (veterinarian/ specialist) advice before prescribing antibiotics?

Mark only one oval.

- Yes
- No

52. 412. Do you use expired antibiotics in the absence of viable antibiotics in your center?

Mark only one oval.

- Yes
- No

53. 413. Do you face shortage of antibiotics in your center

Mark only one oval.

- Yes
- No

54. 414. Drug expiry is a problem in my center

Mark only one oval.

- Yes
- No

55. 415. I give large dose of antibiotics to large animal and small dose to small animal (e.g. Poultry birds) by looking at the size of the animals than the recommended dose

Mark only one oval.

- Yes
- No

56. 416. How do you decide upon whether or not to prescribe an antibiotic for a case? (You can select more than one)

Check all that apply.

- [ ] Based on the previous experience
- [ ] After consultation with other professionals
- [ ] Upon farmers'/owner's request

Thank you for your valuable time in responding to the survey. Kadrinchey La

This content is neither created nor endorsed by Google.

Google Forms
